# Arsenic in Skin Diseases

**Published:** 1869-03

**Authors:** 


Arsenic in Skin Diseases.—Arsenic is of no use whatever in venereal skin diseases, nor in the ephemeral and exanthematous class of affections. It is not useful in the eruptions produced by specific poisons, as many of the forms of lichen; in short, in any eruption which can be traced either to a local or specific cause. With these exceptions, arsenic is almost unexceptionally successful, and in all cases of chronic idiopathic affection of the skin, including lichen simplex, lichen agrius, prurigo, chronic erythema, impetigo, porrigo, alopecia, ecthyma, rupia, lepra, psoriasis, pityriasis, eczema, lupus exedens, etc., provided always, and in all cases, that it be administered discreetly, perseveringly and with such adjuncts (purgatives, alteratives, tonics, or eliminants) as the case may require. It may be added that it is especially useful in those cases of skin disease which depend on uterine irritation or irregularities. (Mr. T. Hunt, in *Braithwaite.*)

